# Analysis of chronic kidney disease patients by targeted next-generation sequencing identifies novel variants in kidney-related genes

**DOI:** 10.3389/fgene.2022.886038

**Published:** 2022-08-11

**Authors:** Manal Alaamery, Jahad Alghamdi, Salam Massadeh, Mona Alsawaji, Nora Aljawini, Nour Albesher, Bader Alghamdi, Mansour Almutairi, Fayez Hejaili, Majid Alfadhel, Batoul Baz, Bader Almuzzaini, Adel F. Almutairi, Mubarak Abdullah, Francisco J. Quintana, Abdullah Sayyari

**Affiliations:** ^1^ Developmental Medicine Department, King Abdullah International Medical Research Center, King Saud Bin Abdulaziz University for Health Sciences, King Abdulaziz Medical City, Ministry of National Guard- Health Affairs, Riyadh, Saudi Arabia; ^2^ Saudi Human Genome Program, National Center for Genomic Technologies and Bioinformatics, King Abdulaziz City for Science and Technology (KACST), Riyadh, Saudi Arabia; ^3^ KACST-BWH Centre of Excellence for Biomedicine, Joint Centres of Excellence Program, King Abdulaziz City for Science and Technology (KACST), Riyadh, Saudi Arabia; ^4^ Saudi Food and Drug Authority, Riyadh, Saudi Arabia; ^5^ Department of Biological Sciences, Faculty of Sciences, King Abdulaziz University, Jeddah, Saudi Arabia; ^6^ Department of Internal Medicine, Division of Nephrology, King Abdulaziz Medical City, Ministry of National Guard- Health Affairs, Riyadh, Saudi Arabia; ^7^ King Saud Bin Abdulaziz University for Health Sciences, Riyadh, Saudi Arabia; ^8^ Medical Genomics Research Department, King Abdullah International Medical Research Center, King Saud Bin Abdulaziz University for Health Sciences, King Abdulaziz Medical City, Ministry of National Guard- Health Affairs, Riyadh, Saudi Arabia; ^9^ Science and Technology Unit, King Abdullah International Medical Research Center, King Saud Bin Abdulaziz University for Health Sciences, King Abdulaziz Medical City, Ministry of National Guard- Health Affairs, Riyadh, Saudi Arabia; ^10^ Department of Medicine, Ministry of the National Guard–Health Affairs, Riyadh, Saudi Arabia; ^11^ Ann Romney Center for Neurologic Diseases, Brigham and Women’s Hospital, Harvard Medical School, Broad Institute of MIT and Harvard, Boston, MA, United States; ^12^ King Abdullah International Medical Research Center, Riyadh, Saudi Arabia

**Keywords:** chronic kidney disease, next-generation sequencing, renal failure, panel sequencing, novel variants

## Abstract

Despite the enormous economic and societal burden of chronic kidney disease (CKD), its pathogenesis remains elusive, impeding specific diagnosis and targeted therapy. Herein, we sought to elucidate the genetic causes of end-stage renal disease (ESRD) and identify genetic variants associated with CKD and related traits in Saudi kidney disease patients. We applied a genetic testing approach using a targeted next-generation sequencing gene panel including 102 genes causative or associated with CKD. A total of 1,098 Saudi participants were recruited for the study, including 534 patients with ESRD and 564 healthy controls. The pre-validated NGS panel was utilized to screen for genetic variants, and then, statistical analysis was conducted to test for associations. The NGS panel revealed 7,225 variants in 102 sequenced genes. Cases had a significantly higher number of confirmed pathogenic variants as classified by the ClinVar database than controls (i.e., individuals with at least one allele of a confirmed pathogenic variant that is associated with CKD; 279 (0.52) vs. 258 (0.45); *p*-value = 0.03). A total of 13 genetic variants were found to be significantly associated with ESRD in *PLCE1*, *CLCN5*, *ATP6V1B1*, *LAMB2*, *INVS*, *FRAS1*, *C5orf42*, *SLC12A3*, *COL4A6*, *SLC3A1*, *RET*, *WNK1*, and *BICC1*, including four novel variants that were not previously reported in any other population. Furthermore, studies are necessary to validate these associations in a larger sample size and among individuals of different ethnic groups.

## Introduction

Chronic kidney disease (CKD) affects over 10% of the adult population worldwide and is now recognized as the most rapidly increasing contributor to the global burden of disease (Thomas et al., 2017). Recent predictions by the Institute for Health Metrics and Evaluation indicate that CKD will become the fifth cause of years of life lost globally by 2040 (Foreman et al., 2018). In Saudi Arabia, end-stage renal disease (ESRD), the terminal manifestation of CKD, affects more than 6% of the population, resulting in substantial morbidity, mortality, and high healthcare costs (Alsuwaida et al., 2010). At present, there are approximately 17,000 patients on dialysis, and this number is increasing at an exponential rate of 8% annually, with a national dialysis incidence of 140 new cases per million population (PMP) (Wang et al., 2016).

Despite the enormous economic and societal burden of CKD, its pathogenesis remains elusive, impeding specific diagnosis and targeted therapy (Romagnani et al., 2017). CKD is a complex disorder comprising numerous pathophysiologically distinct conditions, which share the common feature of leading to persistent anomalies in the kidney structure and/or function (O’Seaghdha and Fox, 2011). Prior research has shown that CKD results from a combination of genetic and environmental factors (Satko and Freedman, 2005). Moreover, familial aggregation of CKD in diverse ethnic groups is well-documented, confirming the role of heritable factors in predisposition to CKD (Cañadas-Garre et al., 2018a). Although CKD can be identified by well-established clinical biomarkers including the estimated glomerular filtration rate (eGFR), albuminuria, or serum creatinine (SCr) levels, the early prediction of individual risk for CKD or the likelihood of later progression to ESRD remains a challenge (Satko and Freedman, 2005; Cañadas-Garre et al., 2018b).

Genetic studies, initially using candidate gene approaches, and more recently, genome-wide association studies (GWAS) have identified numerous genetic biomarkers conferring susceptibility and disease progression in CKD (Köttgen et al., 2012; Sveinbjornsson et al., 2014; Prokop et al., 2018). However, these genetic biomarkers do not account for all the susceptibility to CKD and explain a minority of the overall heritability. Although there are several causes of kidney failure in Saudi patients on dialysis, as reported in the registry of the Saudi Centre for Organ Transplantation (Bullich et al., 2018), genetic and congenital anomalies of the urinary system are listed as the causes of only 2% and 1.6% of cases, respectively. However, this is an underestimated percentage, and many of the causes listed as “unknown” (7%) or hypertension (38%) may well be due to genetic factors. Globally, a 20% prevalence of family history of kidney disease was reported by incident dialysis patients in the USA (of them, 22.2% in diabetes mellitus; 18.9% in hypertension; 22.7% in glomerulonephritis; and 13.0% in patients with other etiologies) (Fallerini et al., 2014). In another US study, 23% of incident dialysis patients had close relatives with ESRD, while 21% of patients over 55 years old had a family history of ESRD. The prevalence values of family history among patients with diabetes, glomerulonephritis, hypertensive nephrosclerosis, and “other” causes of their CKD were 24.4%, 22.5%, 23.2%, and 17.5%, respectively (Riedhammer et al., 2020). Additionally, a study from Norway reported that individuals with a first-degree relative with ESRD had a 7.2 times higher risk of ESRD than individuals without a first-degree relative with ESRD (Cañadas-Garre et al., 2018a). Importantly, heritability studies of eGFR in twin studies reported an estimate of 50% (Cañadas-Garre et al., 2019). The familial aggregation studies demonstrated that heritability ranged from 36 to 75% for eGFR and from 16% to 49% for albuminuria (Cañadas-Garre et al., 2018a; Adam et al., 2020).

In this study, we used a genetic testing approach based on targeted next-generation sequencing of 104 genes causative or associated with CKD in Saudi patients with ESRD. We sought to elucidate the genetic causes of CKD and identify genetic variants associated with CKD and related traits in the Saudi population, supporting precision medicine for Saudi patients with kidney disease.

## Methods

### Ethics statement and study population

Ethical approval for this study and all experimental protocols was obtained from the Institutional Review Board at King Abdullah International Medical Research Center (KAIMRC), Ministry of National Guard—Health Affairs (MNG-HA), and site-specific approvals were obtained from all participating centers. The study and all experimental protocols adhered to the Declaration of Helsinki. All participants or their guardians were consented to by their local team for genetic testing and participation in this study upon recruitment.

### Cohort selection

This study included a total of 1,098 Saudi subjects, including 534 patients with stage 5 CKD who were referred to the Hemodialysis Unit at King Abdulaziz Medical City (KAMC), MNG-HA between September 2019 and March 2020, or the participating centers. The remaining 564 subjects are healthy CKD-free controls whose medical history was retrieved from the MNGHA health database and carefully revised. The inclusion criteria for the cases were as follows: 1) Saudi adults, who are descendants of Saudi parents and grandparents, 2) having CKD stage 5 (including those on dialysis), and 3) being willing to consent to general genetic research. We excluded patients with terminally ill conditions who were unable to provide informed consent.

### Data collection

Detailed demographic and clinical information was collected for all recruited patients from their electronic health records. All extracted data were collected in a predefined and secure database. Collected variables included age, sex, comorbidities, and family history of renal disease. Clinical information included disease manifestations, phenotypic information, and primary causes of ESRD, which were determined by the treating nephrologist.

### Sample collection and preparation

Blood samples were collected in EDTA tubes from all recruited subjects. Genomic DNA was extracted from whole blood using the Gentra Puregene Blood Kit C instrument (QIAGEN, Hilden, Germany) according to the manufacturer’s instructions. The isolated DNA was then quantified using a NanoDropTM spectrophotometer (Thermo Fisher Scientific, Waltham, MA, United States) and/or Qubit fluorometer (Thermo Fisher Scientific, Waltham, MA, United States) using standard procedures. DNA with the A260/280 ratio between 1.8 and 2.0, and A260/230 ratio ≥ was used for NGS library preparation.

### CKD panel curation

We used a predesigned panel that contains 102 genes that have known or suspected associations with CKD. The gene list was curated by a multi-disciplinary team of nephrologists, clinical geneticists, and laboratory scientists (Cyrus et al., 2018). The used panel has shown 57% overall clinical sensitivity, i.e., detection of a likely causal variant that is subsequently confirmed by Sanger sequencing, and the sensitivity values in selected subgroups of patients with glomerular/tubular disorders, cystic kidney diseases, and kidney malformations were 41%, 63%, and 69%, respectively (Cyrus et al., 2018). The complete list of the included genes and description of gene panel design information is provided by the Saudi Mendeliome Group (Cyrus et al., 2018).

### NGS library preparation and sequencing

NGS libraries were constructed using the Ion AmpliSeq Library Kit Plus (Thermo Fisher Scientific, Waltham, MA) according to the manufacturer’s standard protocol. Sequencing was performed on the Ion Chef System instrument (Thermo Fisher Scientific, Waltham, MA) according to the manufacturer’s workflow. A complete library preparation, NGS methodology, and data processing and bioinformatics analysis are described in the SHGP (Cyrus et al., 2018). Additionally, the Ensembl Variant Effect Predictor (VEP, v104) tool was used to annotate all the identified variants using Ensembl release 100 and assembly GRCh37/hg 19 of the human reference genome (Lee et al., 2007). Variants were also compared against the Genome Aggregation Database (GenomAD). Functional assessment of the identified variants was conducted using SIFT (v5.2.2), PolyPhen-2 (v2.2.2), and CADD (v1.6) (Cyrus et al., 2018; O'Seaghdha et al., 2014; Santín et al., 2011).

### Statistical analysis

Baseline characteristics and known risk factors for CKD were summarized by case and control status using frequency (%) and mean (standard deviation). The difference between case and control was compared by the Wilcoxon rank-sum test for continuous variables and by the chi-squared test for categorical variables. The direct association between identified variants and the risk of CKD status was tested by using Fisher’s exact test as implemented in PLINK (Cyrus et al., 2018). A *p*-value that is corrected for multiple comparisons using the Bonferroni method was considered significant for the genetic association test. For all statistical analyses, the significant value is considered at a two-sided 5% level unless stated otherwise.

## Results

### Baseline characteristics

A total of 1,098 participants fulfilled the study eligibility criteria and were included in all subsequent analyses. Of the recruited subjects, 49% were cases (*n* = 534) and the remaining 51% were controls (n = 564). The baseline characteristics of the two groups are shown in Tables 1, 2. There were no significant differences between the two groups in terms of gender or body weight level, but the control group was younger than the case group (44 vs. 59 years; *p*-value <0.05). Existing other comorbidities were more common among cases than in controls, especially for cardiovascular diseases and diabetes (Table 2). The proportion of the common CKD subtypes among cases is provided in Table 3.

**TABLE 1:** Baseline characteristics of the participants.

Variable	Control (*n* = 564)		Case (*n* = 534)		*p*-value
	Mean	SD	Mean	SD	
Age, years	43.9	16.8	59.3	17.7	<0.05
BMI, kg/m^2^	29.5	6.3	29.6	8.1	—
Hemoglobin (Hgb), gm/L	133.8	21.5	111.1	26.9	<0.05
Creatinine, umol/L	76.7	63.3	660.8	308.5	<0.05
eGFR, ml/min/1.73m^2^	102.7	28.8	11	14.8	<0.05
Albumin, g/L	40.2	5.4	38.5	5.0	<0.05
Hemoglobin A1c	5.8	1.2	6.6	1.8	<0.05
Potassium, mmol/L	4.3	0.4	4.8	0.7	<0.05
Sodium, mmol/L	138.2	3.5	134.8	3.9	<0.05
Calcium, mmol/L	2.2	0.16	2.2	0.20	—
Adjusted calcium, mmol/L	2.3	0.11	2.3	0.19	—
Phosphorus, mmol/L	1.2	0.27	1.3	0.53	<0.05
Alkaline phosphatase, U/L	88.6	59.2	148.1	105.9	<0.05
Total bilirubin (T Bili), umol/L	11.9	13.6	11	10.1	—
Uric acid, umol/L	314.3	110.7	335.5	120.5	<0.05
Triglyceride, mmol/L	1.5	3.00	1.5	0.76	—
Cholesterol total, mmol/L	4.7	1.16	4	1.14	<0.05
High-density lipoprotein (HDL), mmol/L	1.2	0.33	1	0.32	<0.05
Low-density lipoprotein (LDL), mmol/L	3.1	1.05	2.3	0.94	<0.05

**TABLE 2:** Categorical baseline characteristics of the participants.

Variable	Control		Case		*p*-value
	Count	%	Count	%	
Gender					
Female	293	52.0	272	50.9	—
Male	271	48.0	262	49.1	—
Age Category					
40 years old or lest	285	50.5	88	16.5	<0.05
41.0 to 60.0	171	30.3	171	32.0	—
61.0 to 80.0	94	16.7	224	41.9	<0.05
81.0 years old or more	14	2.50	51	9.60	<0.05
BMI category					
Underweight	14	2.60	10	7.60	<0.05
Normal	115	21.7	32	24.2	—
Overweight	182	34.4	38	28.8	—
Obese	218	41.2	52	39.4	—
Comorbidity					
Cardiovascular disease	80	16.2	237	49.5	<0.05
Cerebrovascular diseases	37	7.50	179	37.4	<0.05
Diabetes mellitus	24	4.90	274	57.2	<0.05
Hyperlipidemia	143	28.9	159	33.2	—
Hypertension	77	15.6	355	74.1	<0.05
CNS disorders	57	11.5	119	24.8	<0.05
Eye disease	77	15.6	162	33.8	<0.05
Cancer	39	7.90	53	11.1	—

**TABLE 3:** SNPs associated with ESRD.

Gene	SNP^a^	Chr	BP	A	B	MAF		Genotypic frequency (AA/AB/BB)		OR	L95	U95	*p*-value
						Case	Control	Case	Control				
*PLCE1*	rs2274224	10	96039597	C	G	0.40	0.58	89/245/200	205/245/114	0.47	0.40	0.56	5.02E-18
*CLCN5*	rs188947350	23	49807143	A	T	0.04	0.15	0/31/242	0/120/173	0.23	0.15	0.35	1.17E-14
*ATP6V1B1*	rs11681642	2	71163086	C	T	0.25	0.15	31/203/300	28/111/425	1.9	1.53	2.36	3.11E-09
*LAMB2*	rs151037751	3	49160341	C	G	0.004	0.04	0/5/529	0/44/520	0.12	0.046	0.29	1.52E-08
*INVS*	rs61147858	9	102988329	—	TT	0.05	0.01	4/42/488	3/5/556	5.00	2.59	9.65	7.02E-08
*FRAS1*	NR	4	78979258	GTGT	—	0.11	0.05	18/80/436	8/39/517	2.38	1.71	3.32	1.65E-07
*C5orf42*	NR	5	37139483	T	A	0.01	0.05	0/12/522	0/54/510	0.23	0.12	0.43	2.63E-07
*SLC12A3*	rs3214654	16	56927312	A	C	0.06	0.02	7/51/476	6/10/548	3.26	2.00	5.33	5.19E-07
*COL4A6*	rs1266730	X	107448639	G	T	0.29	0.19	35/65/173	17/73/203	1.753	1.394	2.203	1.63E-06
*SLC3A1*	rs3738984	2	44531484	C	T	0.47	0.36	138/221/175	89/233/242	1.518	1.28	1.801	1.80E-06
*RET*	rs2435351	10	43596179	A	G	0.04	0.01	2/44/488	0/13/551	3.956	2.128	7.354	2.47E-06
*WNK1*	rs1951539755	12	974309	C	—	0.21	0.29	35/151/348	56/219/289	0.6297	0.5177	0.766	3.60E-06
*BICC1*	rs2393503	10	60558125	A	T	0.53	0.43	177/207/150	129/230/205	1.457	1.231	1.724	1.28E-05

Abbreviation: A, minor allele; B, major allele; BP, base position; SNP, single nucleotide alteration; Chr, chromosome; MAF, minor allele frequency; OR, odds ratio; L95, lower bond 95% confidence interval; U95, upper bond 95% confidence interval; NR, not reported.

a Functional annotation of the variants is presented in Supplementary Table S5.

### Gene profiling

The NGS panel revealed 7,225 variants in the 104 sequenced genes. More than half of the identified variants are extremely rare variants (MAF <0.001; 52.9%), and only 830 (11.5%) variants were common with an allelic frequency above 0.05 (Supplementary Figure S1). We identified 1,348 (18.7%) new variants that were not previously reported in other databases. These new variants were more common in *FRAS1*, *C5orf42*, *PKD1*, *CEP290*, and *PKHD1* (Supplementary Figure S2, Supplementary Table S1). Most of the variants identified were intronic variants (0.46; Supplementary Table S3), followed by missense variants (0.25). Genes with the highest proportion of possibly or probably damaging variants as predicted by the PolyPhen score (Supplementary Table S4); GRHPR (21%) and FXYD2 (20%); UMOD (19%); LAMB2 (18.2%) and FREM2 (17.4%). Cases had a significantly higher number of the confirmed pathogenic variant as classified by the ClinVar database than control (i.e., individuals with at least one allele of a confirmed pathogenic variant that is associated with CKD; 279 (0.52) vs. 258 (0.45); *p*-value = 0.03).

### SNP association test

Variants with extremely rare allele frequency (MAF <0.001) were excluded from this analysis (n = 3,827 variants), as well as variants that extremely deviated from HWE among the control group only (n = 102 variants); thus, a total of 3,294 variants were included in the association test. Supplementary Table S4 shows the statistically significant SNPs for CKD stage 5 and dialysis. The top three significant SNPs were rs2274224 in *PLCE1* gene (OR = 0.47; 95%CI: 0.39–0.56; *p*-value = 5.02E-18), rs188947350 in *CLCN5* gene (OR = 0.23; 95%CI: 0.15–0.35; *p*-value = 1.17E-14), and rs11681642 in *ATP6V1B1* gene (OR = 1.9; 95%CI: 1.53–2.36; *p*-value = 3.11E-09).

## Discussion

Herein, we screened a relatively large cohort of ESRD patients to identify genetic factors contributing to extremely severe forms of CKD. We applied an advanced genetic testing approach using a targeted NGS gene panel including 102 genes causative or associated with CKD to characterize a cohort of 534 Saudi patients. We identified 13 statistically significant variants within genes implicated in kidney function. All 13 variants were not previously reported to be associated with kidney diseases. Among these 13 variants, four were novel variants that were not previously reported in any other population. Unlike most GWASs that explored the role of genetic factors in kidney function among the general population, this study uniquely reported the association between genetic variants and severe forms of kidney disease among a homogenous disease-specific population.

Several GWASs were performed for variable kidney function traits. The Chronic Kidney Disease Genetic Consortium (CKDGen) performed a GWAS on more than 67,000 individuals of the European ancestry and reported 20 loci associated with renal function and CKD (Prokop et al., 2018). The study showed that a genetic risk score (GRS) including 16 SNPs explained a 1.4% variation in the renal function, as explained by serum creatinine (eGFRcrea). Later GWASs which included larger sample sizes were able to bring the number of variants that are associated with eGFRcrea up to 63, explaining up to 3.99% of the phenotypic variance (Fallerini et al., 2014; Bullich et al., 2018). As expected, variants identified from these GWAS were of modest effect size and common with allele frequency above 5%. Since these studies were performed on the general population with the majority of study participants having eGFR greater than 60 ml/min/1.732, transferring such results into concrete risk estimation, for the development of ESRD remains uncertain. Hence, genotyping these common variants provides minimal clinically relevant information to individual patients. Importantly, Parsa et al. (2013) examined systematically the association of eGFR with common variants in 258 genes responsible for Mendelian forms of kidney abnormalities using the CKDGen Consortium GWAS data [30]. This study, in addition to others, has been largely unsuccessful in identifying common variants in genes that cause rare monogenic forms of CKD, except for a few genes such as *UMOD*, *LPR2*, and *SLC7A4* [30–32]. Although such an approach has proven its success in other complex traits such as diabetes and lipid levels, it suggests that this failure might be a result of relying on the GWAS panel and subjects from the general population [30]. This is specifically true as CKDGen encompasses a general population that may not easily identify new variants beyond what has already been identified by their GWAS approach. These challenges imply that further research is needed by performing targeted sequencing of these genes or whole-exome sequencing in a disease-specific cohort.

As such, our study has targeted patients with ESRD and identified 13 novel genetic variants associated with ESRD in the Saudi population. The top significant variant (NM_016341.4:c.4724G>C) is a missense variant within the *PLCE1* gene that results in the substitution of arginine for proline at position 1,575 (p.R1575P) and is classified by ClinVar as uncertain significance for association with nephrotic syndrome type 3. When the same variant was not reported before for association with nephrotic syndrome type 3, several other variants within *PLCE1* have been reported in familial and sporadic variants within *PLCE1* (O'Seaghdha et al., 2014). The gene product of PLCE1 is expressed in the developing kidney in glomerular podocytes, and sequence alteration may lead to abnormal protein products that cause an arrest in normal glomerular development (O'Seaghdha et al., 2014). The second top variant (NM_001127898.4:c.163 + 72T>A) is a rare intronic variant within the *CLCN5 gene,* 0.00101 allele frequency in gnomAD, which has not been reported before for association with any phenotype. Several variants within *CLCN5* are associated with different types of renal tubular disorders, such as Dent’s disease (Santín et al., 2011). The third variant (NM_001692.4:c.2T>C) causes a missense mutation, resulting in the substitution of methionine for threonine at position 1 (p.M1T) within the *ATP6V1B1* gene, which encodes a component of vacuolar ATPase (V-ATPase), a multisubunit enzyme that mediates acidification of intracellular organelles. *ATP6V1B1* variants are associated with an autosomal recessive form of distal renal tubular acidosis 2 (Santín et al., 2011). In addition, we have identified variants in *LAMB2*, *INVS*, *FRAS1*, *C5orf42*, *SLC12A3*, *COL4A6*, *SLC3A1*, *RET*, *WNK1*, and *BICC1;* which all play major pathophysiological roles in the pathogenesis of kidney functions (Kestilä et al., 1998).

From both clinical and economic viewpoints, understanding the genetic etiology behind CKD is essential before opting for renal transplantation. Such information may provide novel insights into disease recurrence risk post-transplantation. This study was performed in a unique and novel (Philippe et al., 2008) population that has not been previously explored—the uniqueness of the Saudi population lay in the high consanguinity rate—which provides a perfect population to study the genetic basis of diseases. This has been seen previously in a study that detected the highest rate of causative genes in the Saudi population (45.2%) compared to only 13.7% in Al-Hamed et al. (2016), which showed a strong correlation between the rate of consanguinity and the detection rate of disease-causing genes (R2 = 0.9414). Nonetheless, findings from this study might be limited by some important factors; first, the results are reported from a very homogenous population originating from the Saudi population, and it must be replicated in other diverse ethnic groups to validate the reported association. Second, the control group was younger and healthier than the case group, which could confound the reported results. However, given the fact that these genes are implicated in kidney function, they have pathophysiological roles that could mitigate some of these concerns. Third, we have only tested the disease status as a binomial trait, and future studies should be performed to test other kidney-related functions.

In summary, utilizing a comprehensive kidney-disease gene panel using a case-control study in a Saudi Arabian cohort, we identified 13 genetic variants significantly associated with ESRD in this cohort. Since this study was conducted on a disease-specific population and not on a general population, like most of the previously conducted GWAS, it provides more concrete evidence of the roles of these variants in the pathogenesis of ESRD. Furthermore, studies to replicate the findings of this study, as well as functional analysis of the identified variants, may provide measures to reduce the burden of CKD in KSA. In particular, that elucidating the genetic etiology of CKD may have important ramifications when considering disease recurrence risk post-transplantation. Further studies both to replicate these findings in larger sample sizes and among individuals of different ethnic groups and to functionally validate these candidate genetic variants are imperative. This may provide a comprehensive priority list of molecular targets for translational research and eventually help reduce the burden of CKD in KSA (Franceschini et al., 2006).

## Data Availability

Information for existing publicly accessible datasets are contained within the article.
