# Supplementary material for: Analysis of chronic kidney disease patients by targeted next-generation sequencing identifies novel variants in kidney-related genes
Source: Front Genet. 2022 Aug 11;13:886038. doi: 10.3389/fgene.2022.886038 (PMC9407681; doi:10.3389/fgene.2022.886038)
Supplement: Supplementary file 2 [file Presentation1.PPTX]

## Slide 1
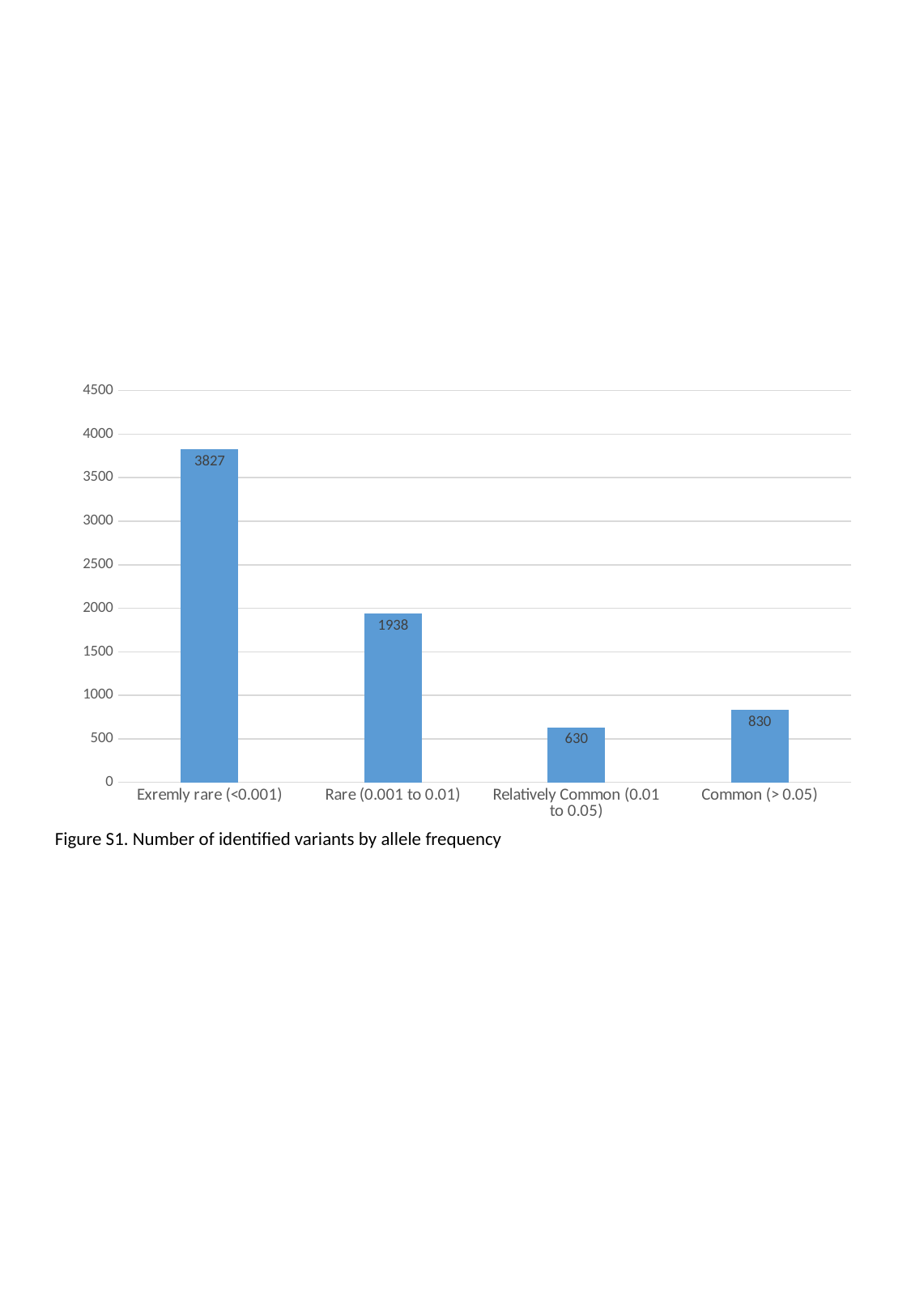

### Chart
| Category | Total |
|---|---|
| Exremly rare (<0.001) | 3827.0 |
| Rare (0.001 to 0.01) | 1938.0 |
| Relatively Common (0.01 to 0.05) | 630.0 |
| Common (> 0.05) | 830.0 |Figure S1. Number of identified variants by allele frequency

## Slide 2
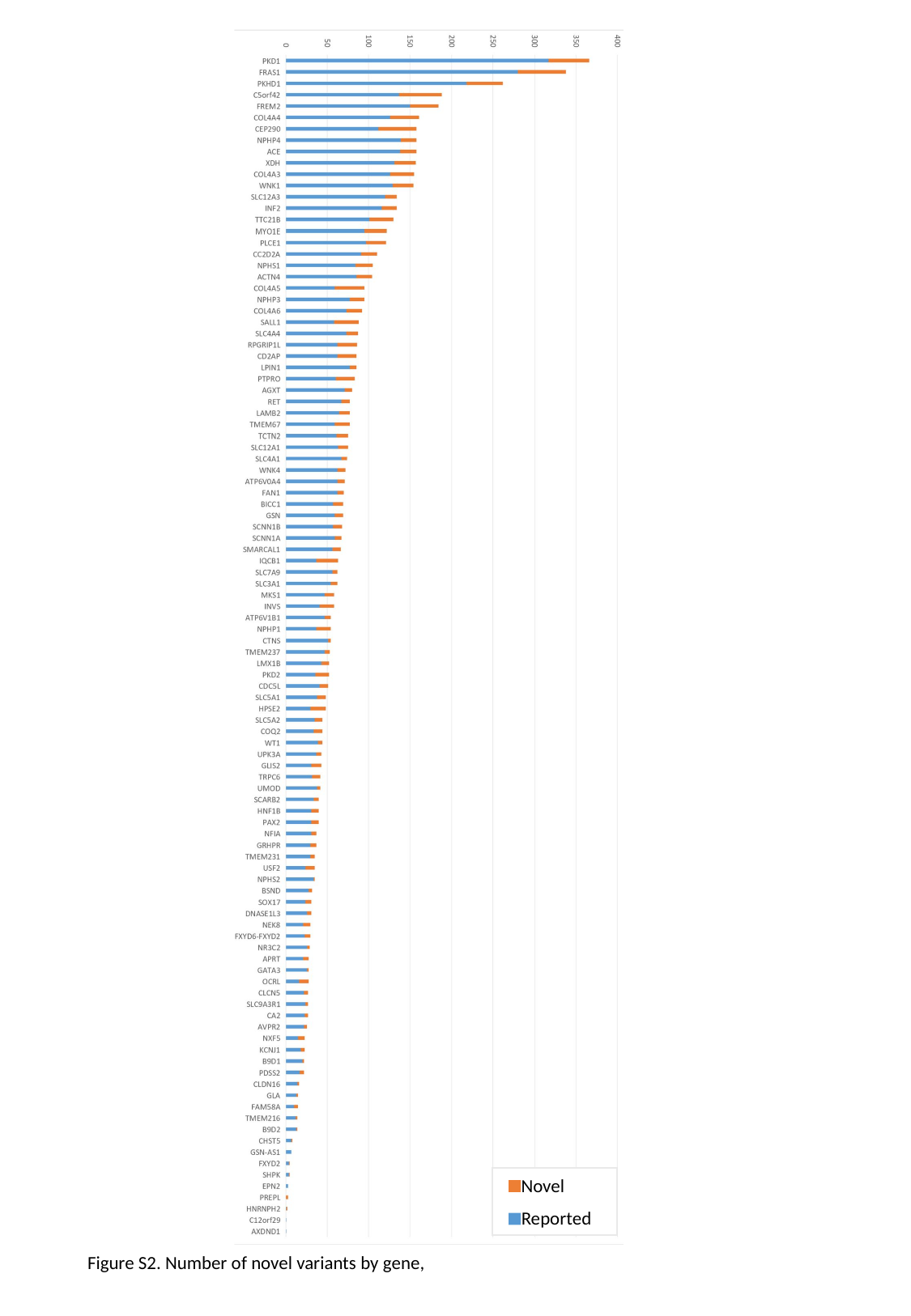

Novel
Reported
Figure S2. Number of novel variants by gene,
