# Supplementary material for: Analysis of chronic kidney disease patients by targeted next-generation sequencing identifies novel variants in kidney-related genes
Source: Front Genet. 2022 Aug 11;13:886038. doi: 10.3389/fgene.2022.886038 (PMC9407681; doi:10.3389/fgene.2022.886038)
Supplement: Supplementary file 3 [file Table2.DOCX]

| gene | chr | gene_semiMergedCDS_size | gene_semiMergedCDS_covered | percent_covered | numSemiMergedCDS_total | numSemiMergedCDS_covered.100.95.90.80.70.50.20..0.0. |
| --- | --- | --- | --- | --- | --- | --- |
| ACE | chr17 | 4120 | 4027 | 0.977 | 26 | 25,0,0,0,0,1,0,0,0 |
| ACTN4 | chr19 | 2736 | 2721 | 0.995 | 21 | 20,0,0,1,0,0,0,0,0 |
| AGXT | chr2 | 1179 | 1179 | 1 | 11 | 11,0,0,0,0,0,0,0,0 |
| APRT | chr16 | 548 | 548 | 1 | 6 | 6,0,0,0,0,0,0,0,0 |
| ATP6V0A4 | chr7 | 2523 | 2444 | 0.969 | 20 | 19,0,0,0,0,0,0,0,1 |
| ATP6V1B1 | chr2 | 1542 | 1542 | 1 | 14 | 14,0,0,0,0,0,0,0,0 |
| AVPR2 | chrX | 2021 | 2021 | 1 | 4 | 4,0,0,0,0,0,0,0,0 |
| B9D1 | chr17 | 1152 | 1152 | 1 | 11 | 11,0,0,0,0,0,0,0,0 |
| B9D2 | chr19 | 528 | 528 | 1 | 3 | 3,0,0,0,0,0,0,0,0 |
| BICC1 | chr10 | 2925 | 2895 | 0.99 | 21 | 20,0,0,1,0,0,0,0,0 |
| BSND | chr1 | 963 | 963 | 1 | 4 | 4,0,0,0,0,0,0,0,0 |
| C5orf42 | chr5 | 9594 | 9530 | 0.993 | 51 | 49,1,0,0,0,0,1,0,0 |
| CA2 | chr8 | 783 | 783 | 1 | 7 | 7,0,0,0,0,0,0,0,0 |
| CC2D2A | chr4 | 5074 | 4985 | 0.982 | 39 | 37,0,0,0,0,1,0,1,0 |
| CD2AP | chr6 | 1920 | 1920 | 1 | 18 | 18,0,0,0,0,0,0,0,0 |
| CDC5L | chr6 | 2409 | 2409 | 1 | 16 | 16,0,0,0,0,0,0,0,0 |
| CEP290 | chr12 | 7440 | 7218 | 0.97 | 53 | 48,1,1,1,0,2,0,0,0 |
| CEP290_hs | chr12 | 12 | 12 | 1 | 2 | 2,0,0,0,0,0,0,0,0 |
| CLCN5 | chrX | 2735 | 2735 | 1 | 15 | 15,0,0,0,0,0,0,0,0 |
| CLDN16 | chr3 | 918 | 918 | 1 | 5 | 5,0,0,0,0,0,0,0,0 |
| COL4A3 | chr2 | 5013 | 4996 | 0.997 | 52 | 51,0,0,1,0,0,0,0,0 |
| COL4A3_hs | chr2 | 14 | 14 | 1 | 2 | 2,0,0,0,0,0,0,0,0 |
| COL4A4 | chr2 | 5073 | 5065 | 0.998 | 47 | 46,0,1,0,0,0,0,0,0 |
| COL4A5 | chrX | 5383 | 5383 | 1 | 53 | 53,0,0,0,0,0,0,0,0 |
| COL4A5_hs | chrX | 28 | 28 | 1 | 4 | 4,0,0,0,0,0,0,0,0 |
| COL4A6 | chrX | 5087 | 5087 | 1 | 46 | 46,0,0,0,0,0,0,0,0 |
| COQ2 | chr4 | 1266 | 1266 | 1 | 7 | 7,0,0,0,0,0,0,0,0 |
| CTNS | chr17 | 1337 | 1337 | 1 | 12 | 12,0,0,0,0,0,0,0,0 |
| CTNS_hs | chr17 | 21 | 21 | 1 | 3 | 3,0,0,0,0,0,0,0,0 |
| DNASE1L3 | chr3 | 918 | 918 | 1 | 8 | 8,0,0,0,0,0,0,0,0 |
| FAM58A | chrX | 775 | 775 | 1 | 6 | 6,0,0,0,0,0,0,0,0 |
| FAN1 | chr15 | 3281 | 3281 | 1 | 14 | 14,0,0,0,0,0,0,0,0 |
| FRAS1 | chr4 | 12305 | 12302 | 1 | 75 | 74,0,1,0,0,0,0,0,0 |
| FREM2 | chr13 | 9510 | 9466 | 0.995 | 24 | 23,0,0,0,0,1,0,0,0 |
| FXYD2 | chr11 | 220 | 220 | 1 | 6 | 6,0,0,0,0,0,0,0,0 |
| GATA3 | chr10 | 1478 | 1461 | 0.988 | 6 | 5,0,1,0,0,0,0,0,0 |
| GLA | chrX | 1290 | 1290 | 1 | 7 | 7,0,0,0,0,0,0,0,0 |
| GLA_hs | chrX | 14 | 14 | 1 | 2 | 2,0,0,0,0,0,0,0,0 |
| GLIS2 | chr16 | 1575 | 1575 | 1 | 6 | 6,0,0,0,0,0,0,0,0 |
| GRHPR | chr9 | 987 | 987 | 1 | 9 | 9,0,0,0,0,0,0,0,0 |
| GSN | chr9 | 2725 | 2725 | 1 | 22 | 22,0,0,0,0,0,0,0,0 |
| HNF1B | chr17 | 1861 | 1861 | 1 | 10 | 10,0,0,0,0,0,0,0,0 |
| HPSE2 | chr10 | 1813 | 1813 | 1 | 13 | 13,0,0,0,0,0,0,0,0 |
| INF2 | chr14 | 3817 | 3707 | 0.971 | 23 | 22,0,0,1,0,0,0,0,0 |
| INVS | chr9 | 3406 | 3397 | 0.997 | 18 | 17,0,1,0,0,0,0,0,0 |
| IQCB1 | chr3 | 1797 | 1797 | 1 | 13 | 13,0,0,0,0,0,0,0,0 |
| KCNJ1 | chr11 | 2325 | 2295 | 0.987 | 4 | 3,0,0,0,0,0,0,0,1 |
| LAMB2 | chr3 | 5397 | 5397 | 1 | 32 | 32,0,0,0,0,0,0,0,0 |
| LMX1B | chr9 | 1453 | 1401 | 0.964 | 10 | 6,0,2,2,0,0,0,0,0 |
| LPIN1 | chr2 | 3227 | 3227 | 1 | 25 | 25,0,0,0,0,0,0,0,0 |
| MKS1 | chr17 | 1730 | 1730 | 1 | 19 | 19,0,0,0,0,0,0,0,0 |
| MT-TL1 | chrM | 75 | 75 | 1 | 1 | 1,0,0,0,0,0,0,0,0 |
| MYO1E | chr15 | 3327 | 3327 | 1 | 28 | 28,0,0,0,0,0,0,0,0 |
| NEK8 | chr17 | 2079 | 2079 | 1 | 15 | 15,0,0,0,0,0,0,0,0 |
| NFIA | chr1 | 1772 | 1772 | 1 | 15 | 15,0,0,0,0,0,0,0,0 |
| NPHP1 | chr2 | 2330 | 2330 | 1 | 22 | 22,0,0,0,0,0,0,0,0 |
| NPHP3 | chr3 | 3993 | 3933 | 0.985 | 27 | 26,0,0,1,0,0,0,0,0 |
| NPHP4 | chr1 | 4281 | 4281 | 1 | 29 | 29,0,0,0,0,0,0,0,0 |
| NPHS1 | chr19 | 3726 | 3668 | 0.984 | 29 | 28,0,0,0,0,0,0,0,1 |
| NPHS2 | chr1 | 1152 | 1129 | 0.98 | 8 | 7,0,1,0,0,0,0,0,0 |
| NR3C2 | chr4 | 2955 | 2955 | 1 | 8 | 8,0,0,0,0,0,0,0,0 |
| NXF5 | chrX | 1098 | 956 | 0.871 | 14 | 12,0,0,0,0,1,0,0,1 |
| OCRL | chrX | 2706 | 2705 | 1 | 24 | 23,1,0,0,0,0,0,0,0 |
| PAX2 | chr10 | 1459 | 1459 | 1 | 13 | 13,0,0,0,0,0,0,0,0 |
| PDSS2 | chr6 | 1200 | 1200 | 1 | 8 | 8,0,0,0,0,0,0,0,0 |
| PKD1 | chr16 | 12962 | 12404 | 0.957 | 47 | 38,4,0,0,4,0,0,0,1 |
| PKD2 | chr4 | 2907 | 2893 | 0.995 | 15 | 14,1,0,0,0,0,0,0,0 |
| PKHD1 | chr6 | 12260 | 12254 | 1 | 67 | 66,0,1,0,0,0,0,0,0 |
| PLCE1 | chr10 | 7191 | 7191 | 1 | 32 | 32,0,0,0,0,0,0,0,0 |
| PTPRO | chr12 | 3655 | 3655 | 1 | 27 | 27,0,0,0,0,0,0,0,0 |
| RET | chr10 | 3525 | 3520 | 0.999 | 21 | 20,0,1,0,0,0,0,0,0 |
| RPGRIP1L | chr16 | 3948 | 3810 | 0.965 | 26 | 25,0,0,0,0,0,0,0,1 |
| SALL1 | chr16 | 7222 | 7222 | 1 | 4 | 4,0,0,0,0,0,0,0,0 |
| SCARB2 | chr4 | 1437 | 1437 | 1 | 12 | 12,0,0,0,0,0,0,0,0 |
| SCNN1A | chr12 | 3088 | 3061 | 0.991 | 15 | 14,0,1,0,0,0,0,0,0 |
| SCNN1B | chr16 | 1923 | 1923 | 1 | 12 | 12,0,0,0,0,0,0,0,0 |
| SLC12A1 | chr15 | 3396 | 3396 | 1 | 27 | 27,0,0,0,0,0,0,0,0 |
| SLC12A3 | chr16 | 3288 | 3288 | 1 | 28 | 28,0,0,0,0,0,0,0,0 |
| SLC12A3_hs | chr16 | 14 | 14 | 1 | 2 | 2,0,0,0,0,0,0,0,0 |
| SLC3A1 | chr2 | 2058 | 2058 | 1 | 10 | 10,0,0,0,0,0,0,0,0 |
| SLC4A1 | chr17 | 2736 | 2660 | 0.972 | 19 | 18,0,0,0,0,1,0,0,0 |
| SLC4A4 | chr4 | 3683 | 3683 | 1 | 27 | 27,0,0,0,0,0,0,0,0 |
| SLC5A1 | chr22 | 2091 | 2091 | 1 | 16 | 16,0,0,0,0,0,0,0,0 |
| SLC5A2 | chr16 | 2019 | 2019 | 1 | 14 | 14,0,0,0,0,0,0,0,0 |
| SLC7A9 | chr19 | 1464 | 1464 | 1 | 12 | 12,0,0,0,0,0,0,0,0 |
| SLC9A3R1 | chr17 | 1077 | 1077 | 1 | 6 | 6,0,0,0,0,0,0,0,0 |
| SMARCAL1 | chr2 | 2865 | 2865 | 1 | 16 | 16,0,0,0,0,0,0,0,0 |
| SOX17 | chr8 | 1245 | 1245 | 1 | 2 | 2,0,0,0,0,0,0,0,0 |
| TCTN2 | chr12 | 2287 | 2287 | 1 | 19 | 19,0,0,0,0,0,0,0,0 |
| TMEM216 | chr11 | 500 | 500 | 1 | 7 | 7,0,0,0,0,0,0,0,0 |
| TMEM231 | chr16 | 1419 | 1418 | 0.999 | 8 | 7,1,0,0,0,0,0,0,0 |
| TMEM237 | chr2 | 1245 | 1203 | 0.966 | 13 | 12,0,0,0,0,0,0,0,1 |
| TMEM67 | chr8 | 3151 | 3101 | 0.984 | 30 | 28,0,0,1,1,0,0,0,0 |
| TRPC6 | chr11 | 2796 | 2796 | 1 | 13 | 13,0,0,0,0,0,0,0,0 |
| TTC21B | chr2 | 3951 | 3927 | 0.994 | 29 | 27,0,0,1,0,1,0,0,0 |
| UMOD | chr16 | 1923 | 1923 | 1 | 10 | 10,0,0,0,0,0,0,0,0 |
| UPK3A | chr22 | 864 | 864 | 1 | 6 | 6,0,0,0,0,0,0,0,0 |
| USF2 | chr19 | 1041 | 989 | 0.95 | 10 | 9,0,0,0,0,0,0,1,0 |
| WNK1 | chr12 | 8848 | 8842 | 0.999 | 32 | 31,1,0,0,0,0,0,0,0 |
| WNK4 | chr17 | 3732 | 3732 | 1 | 19 | 19,0,0,0,0,0,0,0,0 |
| WT1 | chr11 | 1648 | 1641 | 0.996 | 12 | 11,1,0,0,0,0,0,0,0 |
| XDH | chr2 | 4002 | 4002 | 1 | 36 | 36,0,0,0,0,0,0,0,0 |
